# Examining the Impact of LEAD^TM^ Training on Confidence and Practice Patterns in Rehabilitation Professionals

**DOI:** 10.3390/ijerph23070908

**Published:** 2026-07-15

**Authors:** Gedenir Fiorese, Alexa Shinn, Jacklyn Silkes, Katherine S. Judge, Nicole Dawson

**Affiliations:** 1 Doctor of Physical Therapy Program, School of Kinesiology & Rehabilitation Sciences, University of Central Florida, Orlando, FL 32816, USA; 2 Department of Psychology, Cleveland State University, Cleveland, OH 44115, USA

**Keywords:** dementia, confidence, education, training, physical therapy, communication

## Abstract

**Highlights:**

**Public health relevance—How does this work relate to a public health issue?**

- Dementia affects a growing proportion of older adults (≈9.6% of U.S. adults ≥ 65), creating increasing demand for a healthcare workforce prepared to manage its complex functional and communication challenges.
- Rehabilitation professionals play a key role in maintaining function and quality of life in individuals with dementia, yet gaps in training and confidence limit effective care delivery in this population.

**Public health significance—Why is this work of significance to public health?**

- This study addresses a critical workforce gap by demonstrating that targeted training (LEAD™) can significantly improve clinician confidence and adoption of evidence-based dementia care strategies.
- Enhancing provider preparedness has downstream implications for improving care quality, patient safety, and functional outcomes in a rapidly growing and vulnerable population.

**Public health implications—What are the key implications or messages for practitioners, policy makers and/or researchers in public health?**

- Structured, discipline-specific dementia training should be integrated into continuing education to improve confidence and translation of evidence into practice.
- Investment in scalable, rehabilitation-focused dementia education and further research on patient-level outcomes is needed to strengthen the public health response to dementia.

**Abstract:**

Dementia affects approximately 9.6% of Americans aged 65 or older, with these numbers predicted to continue to rise, making it a significant growing public health concern that physical therapists may encounter. Dementia affects a large amount of the PT patient population, yet physical therapists report reduced confidence in treating this population due to the lack of education in academia and reduced exposure to dementia care in clinical practice. This experimental pre-post test study examined the effect of the Leveraging Existing Abilities in Dementia™ training program on confidence in handling various situations and the utilization of communication and treatment strategies discussed in the LEAD™ training program when treating individuals with dementia. Eight rehabilitation providers completed the 12 h LEAD™ training program. Pre-program, post-program, and 3-month surveys were administered to capture confidence in communication, implementation strategies and dementia knowledge. (*p* = 0.003). At baseline, participants were unfamiliar with 5 of 10 validated dementia care practices, but by 3-month follow-up, 90% reported using these strategies at least weekly. Notable gains included intent to use the K.I.S.S. method (+73%) and spaced retrieval (+29%). Although dementia is highly prevalent, many rehabilitation providers lack confidence in dementia care due to limited training. The LEAD™ program shows potential to bridge this gap, but continued evaluation is needed to assess long-term effects on practice and patient outcomes.

## 1. Introduction

Dementia is a progressive neurocognitive disorder that results in the decline of cognitive functioning, including thinking, reasoning, and memory, to a degree that interferes with daily activities and independence. In the United States, dementia affects approximately 9.6% of adults aged 65 years and older, and these numbers are projected to rise substantially with the aging population, positioning dementia as a growing public health concern that healthcare professionals, including rehabilitation professionals such as physical therapists, occupational therapists, and speech-language pathologists, will increasingly encounter (Bennett et al., 2021) [1]. The increasing prevalence underscores the urgent need for an informed, well-prepared rehabilitation workforce capable of addressing the multifaceted challenges associated with dementia care.

Dementia encompasses a group of progressive neurodegenerative disorders that impair memory, communication, cognition, mobility, and participation in meaningful activities, with Alzheimer’s disease representing the most common form (National Institute on Aging, n.d.) [2]. Although rehabilitation professionals play a critical role in maintaining function, promoting safety, and optimizing quality of life for individuals with dementia, many report limited dementia-specific education and low confidence in providing care (Foley et al., 2020; Duarte et al., 2023) [3,4]. Existing dementia education programs have largely targeted nurses, nursing assistants, mental health providers, and other direct care staff, with minimal representation of physical therapists, occupational therapists, or speech-language pathologists (Duarte et al., 2023; Eggenberger et al., 2013; Lewis et al., 2010; Pleasant et al., 2017; Karlin et al., 2014) [3,5,6,7,8]. This educational gap is reflected in the rehabilitation literature. A recent scoping review identified only 67 dementia-related articles among more than 11,000 publications in physiotherapy journals over a 10-year period, suggesting that rehabilitation clinicians often rely on general medical or interdisciplinary literature to guide practice (Omaña et al., 2025) [9]. Similarly, White et al. (2022) [10] identified limited academic preparation, insufficient interprofessional education, and inadequate organizational support as persistent barriers to high-quality dementia care, while Taylor et al. (2024) [11] established an international physiotherapy competency framework that defines the knowledge and skills required for dementia care but does not address how these competencies can be effectively taught and implemented at scale. Collectively, these findings highlight a critical need for scalable, discipline-specific educational programs designed to improve rehabilitation professionals’ knowledge, confidence, and competence in dementia care.

**Figure 1:**
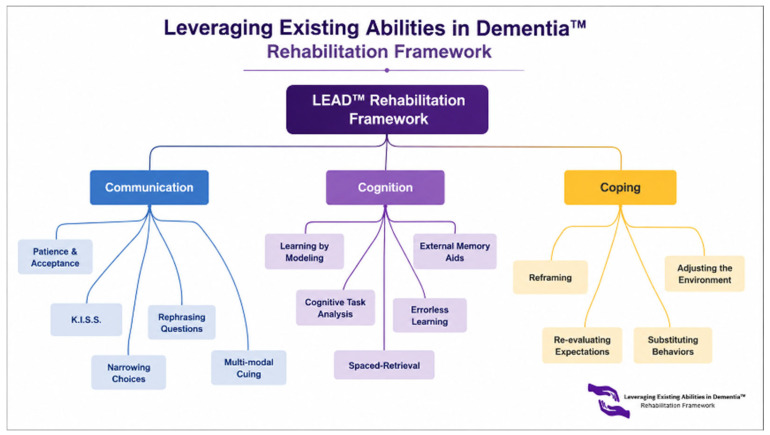
Leveraging Existing Abilities in Dementia^TM^ Rehabilitation Framework showcasing the three C’s—Communication, Cognition, and Coping—with corresponding strategies.

Recognizing this need for specialized dementia training, the Leveraging Existing Abilities in Dementia (LEAD™) Framework (see Figure 1) for Rehabilitation Professionals and its associated training program was developed to enhance rehabilitation professionals’ knowledge, confidence, and use of evidence-based strategies in dementia care (Dawson et al., 2019) [12]. The LEAD™ program adopts a Strength-Based Approach, emphasizing the preservation of existing abilities and capacities rather than focusing solely on cognitive or functional decline. In a previous study of rehabilitation professionals at a home health agency, results revealed significant improvements in participants’ confidence and dementia-related knowledge immediately after training and at three-month follow-up, suggesting that structured educational interventions can positively influence practice behaviors and clinician self-efficacy (Dawson et al., 2019) [12].

In light of these challenges, the current study seeks to expand the evidence supporting the LEAD™ training program by evaluating its implementation among rehabilitation professionals in a Continuous Care Retirement Community (CCRC) setting, which works across various settings on a single campus. Specifically, this study examines whether participation in the LEAD™ program increases providers’ confidence in handling dementia-related situations and enhances the use of communication and treatment strategies with those with dementia. Given the projected rise in dementia prevalence and the ongoing educational deficiencies in rehabilitation professionals’ training and practice, this investigation aims to contribute to the growing body of evidence promoting structured, evidence-informed dementia education for rehabilitation professionals.

## 2. Methods

### 2.1. Study Design

This study used a single-arm non-randomized repeated measures design to assess the impact of the LEAD™ training program on rehabilitation providers’ confidence, knowledge, and use of evidence-based communication and treatment strategies when working with individuals with dementia. Data were collected at three time points, including pre- and post-program surveys and a 3-month follow-up survey. Pre- and post-program surveys were completed in person at the time of training. The 3-month follow-up survey was distributed electronically using Qualtrics (Provo, Utah, XM Platform UI) software (https://www.qualtrics.com/en-au/, accessed on 7 July 2026).

### 2.2. Description of the LEAD^ΤM^ Training Program for Rehabilitation Professionals

The LEAD™ (Leveraging Existing Abilities in Dementia) training program is a structured, two-day (12 h), interdisciplinary educational program for rehabilitation professionals founded on the Strength-Based Approach to dementia care. The curriculum integrates didactic instruction, case-based learning, active learning activities, and evidence-based treatment facilitation strategies to improve clinicians’ knowledge, confidence, and clinical decision-making when working with individuals living with dementia. A comprehensive description of the curriculum, learning objectives, instructional methods, and program development has been published previously (Dawson et al., 2019) [12].

### 2.3. Evaluation of the LEAD^ΤM^ Training Program for Rehabilitation Professionals

#### 2.3.1. Procedure

Attendees were rehabilitation professionals and employees of a CCRC near Jacksonville, FL. Data collection occurred across three time points between February 2025 and June 2025. The initial pre-program and immediate post-program surveys were collected on paper as part of the training evaluation process. Prospective follow-up surveys were distributed electronically through Qualtrics at approximately 3 months after course completion.

Prior to the LEAD™ training program, participants completed a baseline questionnaire that included demographic and professional characteristics, the Dementia Knowledge Assessment Scale (DKAS), the Confidence in Dementia Scale (CODE), and questions regarding their current use of dementia-specific communication and treatment strategies in clinical practice. Immediately following the two-day training program, participants completed the DKAS, the CODE, and a program evaluation questionnaire assessing course acceptability and intended changes in clinical practice. Three months after the training, participants completed the CODE and the practice patterns questionnaire to evaluate sustained changes in confidence and use of dementia-specific treatment strategies.

**Measures**

**Dementia Knowledge Assessment Scale (DKAS)**

Dementia knowledge was assessed using a 27-item true/false version of the Dementia Knowledge Assessment Scale (DKAS) (Annear et al., 2015) [13]. Items assess knowledge related to dementia etiology, disease progression, communication, behavioral symptoms, care considerations, and risk reduction. Total scores range from **0 to 27**, with higher scores indicating greater dementia knowledge. Sample items include “Exercise is generally beneficial for people experiencing dementia” and “People with advanced dementia often communicate through body language”. The original DKAS has demonstrated excellent psychometric properties, including high internal consistency (Cronbach’s α = 0.89) and evidence of construct, concurrent, factorial, and test–retest reliability (Annear et al., 2015) [13].

**Confidence in Dementia Scale (CODE)**

Confidence in working with individuals with dementia was assessed using the Confidence in Dementia Scale (CODE) (Elvish et al., 2018; Lorio et al., 2017) [14,15]. The CODE is a nine-item, unidimensional measure of clinicians’ confidence when managing common dementia care situations. Items are rated on a 5-point Likert scale, with higher scores indicating greater confidence. Sample items include confidence in effectively communicating with individuals living with dementia and managing challenging dementia-related situations. The CODE has demonstrated excellent internal consistency (Cronbach’s α = 0.91), established construct validity, and responsiveness to dementia education interventions.

**Practice Patterns Questionnaire**

Clinical practice patterns were evaluated using a study-specific questionnaire originally developed for the LEAD™ training program. The questionnaire assessed the frequency with which participants used evidence-based dementia communication and treatment strategies in routine clinical practice, including validation, keeping instructions short and simple (KISS), reframing behaviors, narrowing choices, multimodal cueing, spaced retrieval, cognitive task analysis, errorless learning, and learning by modeling. Participants rated each strategy using a 6-point Likert scale ranging from **0 = I don’t know that method** to **5 = Daily**. Immediately following the program, participants reported their intended frequency of use, whereas the three-month follow-up assessed actual implementation in clinical practice.

**Program Acceptability**

Immediately following the training, participants completed a program evaluation questionnaire assessing overall acceptability of the LEAD™ training program. Participants rated instructor effectiveness, organization of course content, relevance to clinical practice, achievement of learning objectives, and usefulness of instructional materials using a 5-point Likert scale ranging from **1 = Poor** to **5 = Excellent**. These items were developed specifically to evaluate participant satisfaction with the LEAD™ training program and were adapted from the original pilot evaluation.

#### 2.3.2. Statistical Analyses

Data were analyzed using JASP statistical software (version 0.19.3 Intel). Descriptive statistics were used to summarize sample characteristics. Frequencies were calculated to determine the level of endorsement for survey items related to practice patterns. A paired-samples *t*-test was conducted to evaluate changes in dementia knowledge. A repeated-measures ANOVA was conducted to examine whether there were significant differences in confidence in working with people with dementia at time 1, time 2 and time 3.

## 3. Results

### 3.1. Participants

Eight rehabilitation providers participated in the study and completed the 12 h LEAD™ training program. Participants included licensed clinicians working in rehabilitation settings, such as physical therapists, occupational therapists, and speech-language pathologists. Prior to program participation, attendees provided demographic information, including professional designation, highest educational degree, years of practice, and previous experience working with individuals with dementia.

All participants were recruited from Vicar’s Landing, a Continuous Care Retirement Community (CCRC) located in Ponte Vedra Beach, Florida, where the LEAD™ training program was implemented. The sample consisted of four physical therapists or physical therapist assistants, three occupational therapists or certified occupational therapy assistants, and one speech-language pathologist. Educational backgrounds varied: three participants completed two years of formal education to attain their degree, four completed three years, and one completed four years. On average, participants had 22.5 years of experience working in healthcare and 20.2 years of experience working with individuals with dementia. Prior to completing the LEAD™ program, participants reported an average of 16.2 h of previous dementia-specific training. Seven of the eight participants held two dementia-specific certifications, and one participant held one dementia-specific certification.

### 3.2. Pre-Post Comparison of Dependent Measures

**Figure 2:**
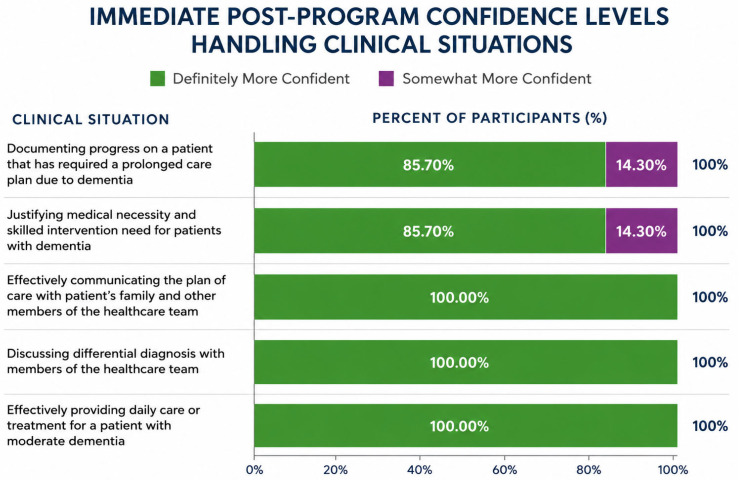
Immediate post-program confidence levels in handling clinical situations. Note: Survey question: How confident are you in the following situations? Answers rated using a 5-point Likert scale (1 being “Feel Less Confident” and 5 being “Definitely More Confident”).

Some outcomes demonstrated improvements, while others remained constant. Paired sample *t*-tests and descriptive analysis showed that scores on the DKAS remained relatively constant between pre- and post-program testing, with a pre-program mean of 22.6 (max 27) and post-program mean of 22.3 (*t* = 0.424, *p* = 0.685), indicating attendees were selecting similar answers regarding dementia knowledge pre- and post-program. Following the LEAD™ training program, attendees demonstrated significant increases in practice confidence, as measured on the CODE, involving handling situations, communication techniques and treatment strategies for those with dementia, from a mean of 49.7 (max 70) to 69.1 at post-test (*p* < 0.001). Specifically, 100% of attendees were “definitely more confident” in providing daily treatment to those with moderate dementia, discussing differential diagnosis with members of the healthcare team, and effectively communicating the plan of care to the patient’s family and 85.7% (7/8) were “definitely more confident” in justifying medical necessity and skilled intervention for patients with dementia and documenting progress on a patient who requires a prolonged care plan due to dementia following participation in the LEAD™ program (see Figure 2).

**Figure 3:**
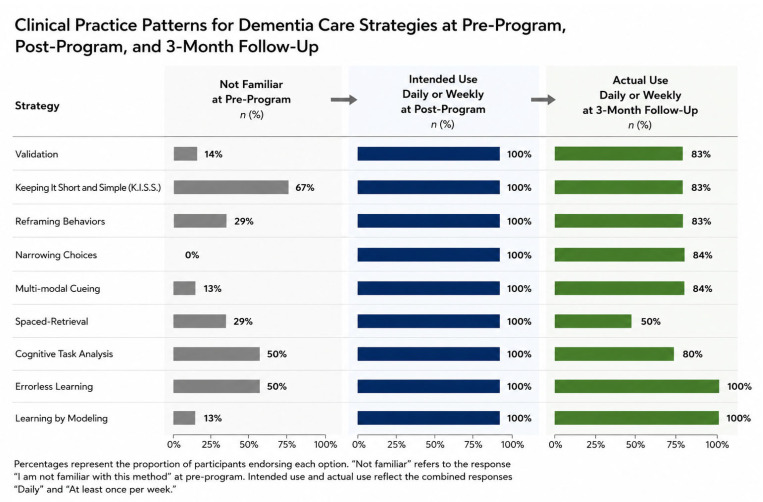
Frequency of using communication and treatment strategies. Familiarity (pre-program), intended use (post-program), and actual use (3-month follow-up). Note: Survey question: How often do you use the following communication and treatment strategies? Answers rated using a 5-point Likert scale (0 being “I am not familiar” and 5 being “Daily”).

Additional findings showed that despite previous dementia-specific education and certifications, participants were unfamiliar with 9/10 evidence-based dementia care practice patterns, which include tailored communication and treatment strategies prior to the LEAD™ training program (Figure 3). The “Keeping it Short and Simple” (K.I.S.S.) strategy was deemed the most unfamiliar, with 67% (6/8) of participants marking “I am not familiar” when asked how often they used this method prior to LEAD™. Following implementation of LEAD™, all attendees showcased planning to include the communication and treatment strategies covered in the program. All responses showcased the intent to implement these practice patterns either “daily” or “at least once a week”, with 6/10 strategies at 100% for daily usage and the remaining strategies ranging from 75 to 88% for intent to use daily.

### 3.3. Three-Month Follow-Up Measure

**Figure 4:**
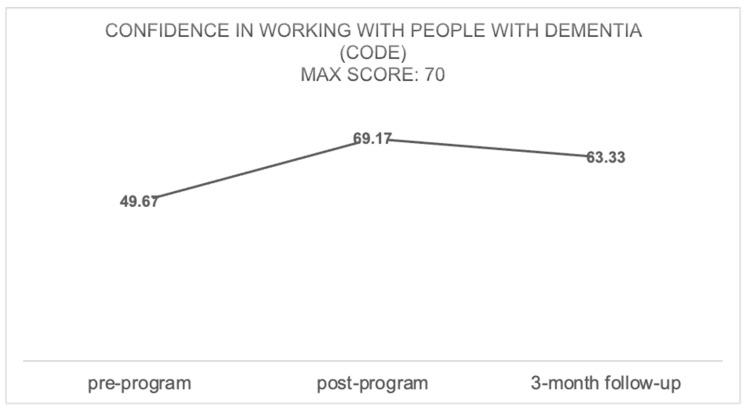
Confidence in working with people with dementia (CODE) mean scores at pre-game, post-program, and 3-month follow-up.

Of the eight attendees at the LEAD™ training program, six (75%) responded to the 3-month follow-up survey. A repeated measures ANOVA was conducted to examine whether there were significant differences in confidence in working with people with dementia at time 1, time 2 and time 3 (see Figure 4). The data met assumptions of Mauchly’s test of sphericity (*p* = 0.724); therefore, no adjustments were needed in analyses. There was a significant main effect of time for the CODE overall score F_(2,10)_ = 15.469, *p* < 0.001, *η*_p_^2^ = 0.756, indicating a very large effect size. Post hoc pairwise comparisons were conducted to investigate whether differences between the means occurred between T1 and T2, or T2 and T3. The comparisons indicated that there was a significant difference between the average score, which was lower at T1 ($\bar{x}$ = 49.67 (9.771)) than T2 ($\bar{x}$ = 69.17 (1.329)) (*p* = 0.015). There was no significant difference between T2 and T3 ($\bar{x}$ = 63.33 (6.919)) (*p* = 0.296), suggesting sustained improvements in confidence at the 3-month follow-up. Attendees continued to report the frequency of using dementia care practice patterns (see Figure 3) as either “occasionally”, “at least once a week” or “daily” to showcase that the use of these evidence-based strategies was sustained throughout the 3-month follow-up. No attendee ever reported “never” using these learned techniques or “I don’t know that method” on the 3-month follow-up survey.

## 4. Discussion

Findings from the LEAD™ training program suggest meaningful improvements in participant confidence and implementation of evidence-based dementia care strategies into daily practice, even though dementia knowledge scores (DKAS) remained relatively stable pre- and post-testing. The significant gains in practice confidence (*p* = 0.003) indicate that the LEAD^TM^ training program effectively enhanced attendees’ self-efficacy in managing complex situations, communication strategies, and treatment planning for individuals with dementia despite their prior experiences and training in this area. These improvements reflect an important outcome, as confidence is a key precursor to behavior change and clinical skill application. Furthermore, post-program responses demonstrate a strong intention to adopt validated dementia care strategies in daily practice, reflecting meaningful translation of training content into anticipated clinical behavior. The 3-month follow-up responses revealed sustained improvements in confidence along with daily and weekly use of evidence-based treatment strategies.

The findings of the present study can be interpreted within the context of the Kirkpatrick Model, one of the most widely applied frameworks for evaluating educational interventions in health professions education (Kirkpatrick & Kirkpatrick, 2006) [16]. The LEAD™ training program demonstrated positive outcomes across the first three levels of the model. Participants reported high satisfaction with the training (Level 1: Reaction), demonstrated significant improvements in confidence when managing individuals living with dementia (Level 2: Learning), and reported sustained implementation of evidence-based dementia care strategies three months following the program (Level 3: Behavior). As noted by Yardley and Dornan (2012) [17], educational evaluations are strengthened by examining outcomes beyond learner satisfaction to include meaningful changes in professional practice. The sustained changes in clinicians’ reported practice patterns suggest that the LEAD™ training program facilitated translation of learning into clinical behavior. Future research should extend this work by evaluating Level 4 outcomes, including the impact of clinician training on patient participation, functional outcomes, and quality of care.

Although dementia knowledge as measured by the DKAS did not change significantly following the LEAD™ training program, participants demonstrated substantial improvements in confidence when managing individuals living with dementia and reported sustained implementation of evidence-based dementia care strategies. At baseline, participants represented an experienced rehabilitation workforce, averaging nearly two decades of dementia care experience, with many reporting previous dementia-related continuing education and certifications. Despite this experience, participants reported only moderate confidence in delivering dementia-specific rehabilitation and limited familiarity with several evidence-based treatment strategies, including K.I.S.S., spaced retrieval, cognitive task analysis, and errorless learning. These findings suggest that prior dementia education may provide foundational knowledge but does not necessarily equip rehabilitation professionals with the discipline-specific clinical skills and strategies needed to confidently deliver person-centered rehabilitation. Rather than focusing on general dementia knowledge, the LEAD™ training program was designed to translate existing knowledge into practical, rehabilitation-specific clinical decision-making and intervention strategies. The large improvement in clinician confidence (η^2^ = 0.756), coupled with sustained changes in reported clinical practice, suggests that targeted rehabilitation-specific education may address an important gap not filled by more general dementia education programs.

This supports current literature that suggests many current dementia-specific staff training programs focus on nursing staff and direct service caregivers. While these caregiver-focused programs show benefits in knowledge, confidence, stress reduction, and behavioral outcomes, they do not address the unique therapeutic decision-making, motor learning principles, and functional outcome targets central to rehabilitation practice. This gap leaves rehabilitation professionals without accessible, discipline-specific tools tailored to the demands of their clinical roles. The LEAD^TM^ Framework for Rehabilitation Professionals and its associated training program have great potential to meet this need as they incorporate all five key domains outlined in the e-Delphi study. Rehabilitation professionals demonstrated improved confidence in their ability to manage and treat this complex population and adopted sustained use of evidence-based treatment strategies following their training.

While these results contribute significantly to the current body of literature on dementia training in rehabilitation professionals, the reader should consider a few limitations when interpreting these findings. The small sample size represents an important limitation and restricts the generalizability of these findings. As an initial evaluation of the LEAD™ training program, the study was designed to examine preliminary changes in clinician knowledge, confidence, and attitudes following participation. Future research involving larger, more diverse cohorts, including participants from multiple disciplines, organizations, and geographic regions, is needed to establish the robustness and external validity of these findings. Ongoing implementation of the LEAD™ training program through larger dissemination efforts will provide opportunities to evaluate these outcomes in substantially larger samples. The three-month follow-up was conducted electronically following participants’ return to clinical practice, whereas the baseline and immediate post-program assessments were completed in person. This difference in survey administration, combined with the lower follow-up response rate, may have introduced response bias and limits interpretation of the longer-term findings. Another limitation is the use of a single-group pre-post design without a comparison group. Although this design is commonly used to evaluate immediate educational outcomes, it precludes attributing observed changes exclusively to the LEAD™ training program. However, given the brief interval between pre- and post-assessments and the absence of other educational interventions during that period, alternative explanations for the observed improvements are less likely. Future studies employing controlled or randomized designs would strengthen causal inferences regarding program effectiveness. A final limitation for the reader to consider is the reliance on self-report outcome measures. Although validated instruments were used where available, participants may have overestimated improvements in knowledge, confidence, or attitudes due to social desirability or expectancy effects. Future studies should incorporate objective measures of clinician behavior, knowledge retention, and patient outcomes to complement self-reported outcomes.

Additionally, future directions of research in this area should include patient-level outcomes (e.g., Kirkpatrick model Level 4, transitions in level of care, aging in place versus institutionalization, falls, behaviors) to determine if the LEAD^TM^ training program leads to improved patient outcomes. Translating the training program to an online learning platform to allow accessibility to a wider population of rehabilitation professionals would be prudent.

In summary, the LEAD™ training program demonstrated meaningful improvements in provider confidence and regular use of evidence-based dementia care strategies in service provision, reinforcing the importance of structured, interdisciplinary education in this area. Although dementia knowledge scores remained stable, the program successfully enhanced attendees’ perceived competence in clinical decision-making, communication, and treatment planning for individuals with dementia. These findings suggest that confidence-building and skill reinforcement may be key mechanisms for translating training into improved patient outcomes. The LEAD^TM^ training program directly addresses a long-standing and critical gap in the literature: the near absence of dementia-specific training programs designed for and tested with physical therapists, occupational therapists, and speech-language pathologists. Whereas most existing programs target nurses, aides, or family caregivers, rehabilitation providers are seldom included, despite their central role in restoring function, promoting mobility, and reducing disability for people living with ADRD. By centering the needs, competencies, and practice realities of rehabilitation professionals, this study represents a needed contribution to the dementia care landscape. Moreover, effective dementia rehabilitation requires a collaborative, team-based approach in which PT, OT, and SLP professionals integrate their complementary expertise to optimize patient outcomes.

## Data Availability

The raw data supporting the conclusions of this article will be made available by the authors on request.
